# From bench to bar side: Evaluating the *red wine storage lesion*

**DOI:** 10.1515/biol-2021-0089

**Published:** 2021-08-28

**Authors:** Sven Klaschik, Richard K. Ellerkmann, Jennifer Gehlen, Stilla Frede, Tobias Hilbert

**Affiliations:** Department of Anesthesiology and Intensive Care Medicine, University Hospital Bonn, Venusberg-Campus 1, 53127 Bonn, Germany; Department of Anesthesiology and Intensive Care Medicine, Dortmund Hospital, Beurhausstrasse 40, 44137 Dortmund, Germany

**Keywords:** transfusion, perioperative care, wine, resveratrol, antioxidants, Hep G2 cells

## Abstract

Vitally essential red fluids like packed cells and red wine are seriously influenced in quality when stored over prolonged periods. In the case of red cell concentrates, the resulting *storage lesion* has particular significance in perioperative medicine. We hypothesized that, in contrast, aging rather improves the properties of *red wine* in several ways. A translational approach, including (I) *in vitro* experiments, (II) a randomized, blinded crossover trial of acute clinical effects, and (III) a standardized red wine blind tasting was used. Three monovarietal wines (Cabernet Sauvignon, Chianti, Shiraz) in three different vintages (range 2004–2016), each 5 years different, were assessed. Assessments were performed at a German university hospital (I, II) and on a garden terrace during a mild summer evening (III). Young wines induced cell stress and damage while significantly reducing cytoprotective proteins in HepG2 hepatoma cells. Sympathetic activity and multitasking skills were altered depending on wines’ ages. Hangovers tended to be aggravated by young red wine. Aged variants performed better in terms of aroma and overall quality but worse in optical appearance. We found no evidence for a *red wine storage lesion*. However, we plead for consensus-based guidelines for proper storage, as it is common in clinical medicine.

## 1 Introduction

Age matters, as every health professional knows. Recently, the effect of aging on biochemical and physical properties of packed red blood cells (PRBCs) has gained more attention, not only in perioperative medicine. This *red blood cell storage lesion* is supposed to impact transfusion outcomes significantly [1]. Consequently, due to a growing shortage of red cell concentrates, the *Patient Blood Management* (PBM) program aims to reduce PRBC consumption (for additional information, please also visit www.patientbloodmanagement.de) [2].

Not at least for physicians, *red wine* may be as vitally important as red blood. Ernest Hemingway stated: ‘Drinking wine […] was as natural as eating and to me as necessary’ [3]. However, unlike PRBCs, this red fluid – not less appreciated by a doctor than blood products [4] – if stored properly, is supposed to increase in quality during aging [5]. Although it is a current subject of intense debate [6], wine enthusiasts emphasize red wine’s health-promoting properties – in addition to culinary aspects – to legitimize alcohol consumption. The assumed positive effects range from improving cardiovascular health to preventing cancer and even sunburn [7,8]. This culminates in the fact that red wine, regularly and moderately consumed during meals, explains the so-called *French paradox*. The concept, introduced by its “father” Serge Renaud in 1991, describes that, in France, despite a considerable consumption of saturated fatty acids, a relatively low cardiovascular mortality rate is observed [9]. With nearly 1.8 billion bottles opened in 2013 in France alone (making it the top red wine-consuming country in Europe), these effects are of utmost importance from the health economics point of view [10]. Therefore, it is all the more surprising that the aging of red wine has not yet been addressed scientifically in any way regarding those medicinal effects.

Our study, involving wines made of various varietals, is the first to explore the impact of aging on red wines’ biochemical and culinary characteristics and the aspects of its clinical significance. The question that preoccupied us was: do we have to fear the *red wine storage lesion*?

## 2 Material and methods

### 2.1 Wine selection

Three different wine varietals made from grape varieties from the same vineyard and grown at the same site were analyzed to assess the influence of aging. For that, each varietal was obtained from three different vintages, each with 5 years differences (nine wines in total; see Table 1):

− Cabernet Sauvignon (CS) (Los Vascos (Domaines Barons de Rothschild [Lafite]), Colchagua Valley, Chile): 2016, 2011, 2006
− Chianti/Sangiovese (Ch) (Rúfina Nipozzano Riserva [Frescobaldi], Tuscany, Italy): 2014, 2009, 2004
− Shiraz (Sh) (Neethlingshof Estate, Stellenbosch, South Africa): 2014, 2009, 2004.

**Table 1:** Basic and biochemical characteristics of the nine different tested wines

	Cabernet Sauvignon			Chianti			Shiraz		
	2016	2011	2006	2014	2009	2004	2014	2009	2004
Outward appearance	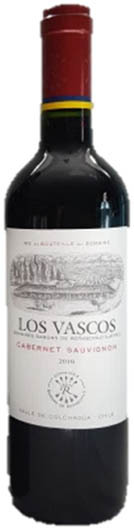	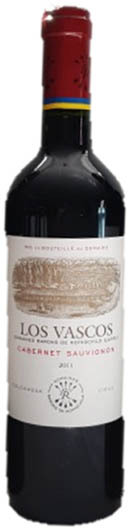	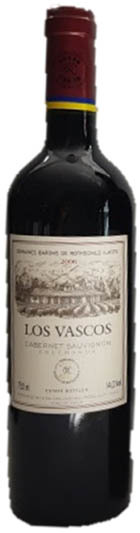	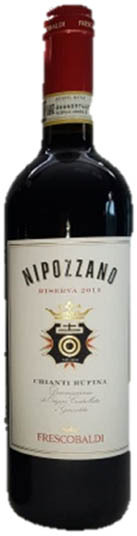	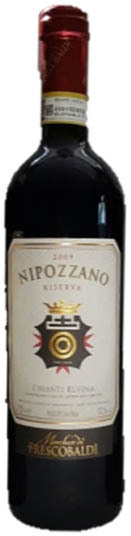	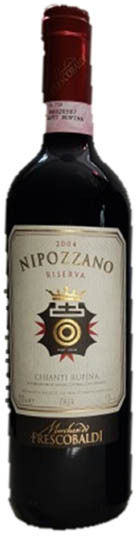	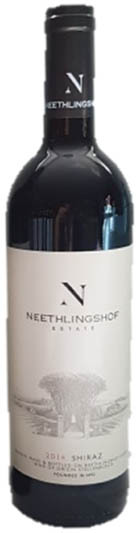	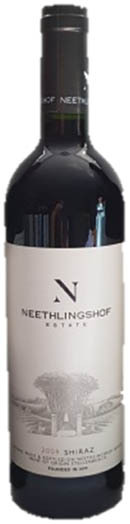	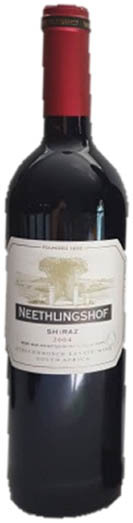
Country of origin	Chile			Italy			South Africa		
Designation, winery	Los Vascos (Domaines Barons de Rothschild [Lafite]), Colchagua Valley			Rúfina Nipozzano Riserva [Frescobaldi], Tuscany			Neethlingshof Estate, Stellenbosch		
Alcohol content (vol%)*	14.0	14.0	14.0	13.0	13.5	13.0	14.0	14.5	14.5
Type of closure	Plastic stopper			Single-piece natural cork			Agglomerated cork		
pH value	3.28 *(0.003)*	3.37 *(0)*	3.43 *(0.003)*	3.4 *(0.003)*	3.3 *(0.003)*	3.45 *(0)*	3.51 *(0.003)*	3.51 *(0.003)*	3.58 *(0.012)*
Resveratrol content (µg/mL)	13.6 *(0.09)*	15.5 *(0.42)*	n.d.	11.8 *(2.98)*	n.d.	n.d.	12.1 *(0.4)*	13.8 *(0.23)*	16.6 *(1.38)*

*Alcohol content according to manufacturer’s specifications.

pH and resveratrol values are given as mean from replicates ± SEM (in brackets); n.d. = not determinable (certain ingredients of the old and middle-aged variants of Cabernet Sauvignon and Chianti seemed to interfere with the detection method or with resveratrol itself, making quantification impossible).

Regions with stable climates were chosen to minimize the influence of climate change. No blended wines but only monovarietal wines were selected to avoid any possible bias due to changing composition over the years. All test substances were purchased from professional German retailers in order to warrant proper storage conditions from date of production (Oberhuber [Munich], Vinumnobile [Reutlingen], GVG Genuss GmbH [Mülheim am Main], Bredick’s [Meckenheim], Perbaccowein [Hanover], Classic Weinkontor [Starnberg], and Wein-Bischoff [Nuremberg]). Before the assessment, all bottles were stored in a dry, dark, and cool environment in a horizontal position.

### 2.2 Aging assessment

Effects of red wine aging were assessed using the following translational approach:

− biochemical aspects: *in vitro* analyses of biological effects on HepG2 cell culture,
− clinical aspects: impact on hemodynamics, metabolism, mental performance and development of headache after acute red wine ingestion by healthy volunteers (Figure 2), and
− culinary aspects: a structured blind tasting that involved wine ingestion by a stratified sample consisted of both laypersons and experts.

#### 2.2.1 *In vitro* analysis

Resveratrol (RVT) was quantified in red wines using a commercially available competitive inhibition ELISA kit (Biozol [Eching, Germany]) according to the manufacturer’s protocol. Results are given in µg/mL. Wine pH was assessed using a Beckman Coulter 340 pH meter (Brea, CA, USA). The human hepatoma cell line HepG2, obtained from American Type Culture Collection (ATCC HB 8065; Manassas, VA, USA), was grown in RPMI 1640 (Life Technologies [Carlsbad, CA, USA]) supplemented with 10% fetal calf serum (Life Technologies) in a humidified atmosphere (5% CO_2_ in air) at 37°C. At the beginning of each experiment, subconfluent HepG2 cells received fresh medium supplemented with the respective red wines. Cell culture medium alone was used as a negative control. Initial dose titrations, including an ethanol control, revealed optimal experimental conditions and no direct cytotoxic effects when incubating cells with 2.5 vol% wine in a cell culture medium. After incubation for 24 h, the supernatant was collected, cells were washed and lysed, and total protein and RNA were extracted using RIPA buffer and the TRIzol™ reagent method (Life Technologies), respectively, according to standard protocols.

In cell supernatant, alanine aminotransferase (ALT), aspartate aminotransferase (AAT), and glutamate dehydrogenase (GLDH) were determined using VIS photometry. Following cDNA synthesis using the High Capacity cDNA Reverse Transcription Kit (Applied Biosystems [Weiterstadt, Germany]), gene expression analysis of interleukin 8 (IL-8) was performed by quantitative real-time PCR (RT-PCR) with Taqman™ Expression Assay and Taqman™ Gene Expression Master Mix on a ViiA7 device (all Applied Biosystems). Expression was normalized to 18 s ribosomal RNA as house-keeping gene and calculated as fold change expression of the respective control using the delta–delta CT method (RQ, relative quantification).

Protein arrays were performed using the Proteome Profiler Human Cell Stress Array Kit (R&D Systems [Wiesbaden-Nordenstadt, Germany]). In short, HepG2 cell lysates were incubated with the arrays at 4°C overnight, and proteins were detected and analyzed according to the manufacturer’s instructions using the Amersham Imager 600 (GE Healthcare [Chicago, IL, USA]).

#### 2.2.2 Clinical assessment

The short-term influence of red wine consumption on hemodynamics and sympathetic activity has been previously described [11]. Thus, effects of red wines of different ages were assessed in three healthy male volunteers without any known previous health issues and with no varying dietary habits (age 39–47 years) using a randomized, blinded crossover design (see CONSORT flow chart, Figure 2a).

Participants were instructed to fast at least 6 h before the assessment and to refrain from consuming caffeine, wine, or grape juice to ensure inter-individual comparability and stable baseline conditions. They were then randomly assigned to either the old wine, middle-aged wine, or young wine group. They were not told which group they were assigned. Bodyweight and height were measured. The following parameters were assessed at baseline before ingestion of test substances with the participants lying in the supine position (Figure 2b):

− heart rate (HR) and mean arterial blood pressure (MAP; M540 Infinity™ monitor, Draeger [Lübeck, Germany])
− serum sampling to detect serum RVT levels (determined by ELISA, see above). Serum samples were stored at −80°C until being processed.
− psychomotor coordination and vigilance.

The participants performed three standardized app-based tests to assess their psychomotor coordination capabilities and their vigilance level. All tests are originally used to detect the cognitive performance of those diagnosed with sleep disorders. They have previously been validated for the assessment of acute alcohol effects [12]. These tests were performed on a Galaxy S8 Plus smartphone (Samsung Electronics Co., Ltd. [Suwon, South Korea]) with the test persons seated on a chair and holding the device in their hands. In the “Divided Attention Test” (DAT), the participant keeps a finger inside a circle that moves over the display, while at the same time, a bar has to be balanced within a moving one-centimeter area at the upper end of the display by tilting the device left or right. Three trials were conducted for each person. The time needed to complete each trial was measured in seconds, with a longer duration indicating better performance. In the “Vigilance Test” (VIG), a spot moves clockwise in single steps and regular positions at one-second increments over the display. The participant touches a button whenever the spot skips a position. This test took 5 min to complete, and the percentage of correctly detected skipped positions was taken. In the “Digit Span Test” (DST), which is a measure of working memory, the test person is shown a defined series of digits (starting with four digits) which he has to reproduce. The number of presented digits increases until the participant fails. Each person was given three trials, and the highest span of correctly retrieved digits was noted.

Following the assessment of baseline parameters, each participant has consumed a standardized amount of red wine. It was previously shown that two ‘standard drinks’ (i.e., two 4-oz glasses of red wine (120 mL each)) might provoke significant clinical effects in an adult test subject [13]. CS, Ch, and Sh of the respective vintage were blended in equal proportions, and each participant had to drink 4.5 mL per kg bodyweight of old, middle-aged, or young red wine cuvée within 10 min, respectively.

HR and blood pressure were taken 30, 60, and 120 min after that. Serum for resveratrol levels was sampled 30 min following red wine intake. Tests for psychomotor coordination and vigilance were performed 30 and 60 min after wine consumption. On the following morning, the intensity of the headache was ascertained using the Numeric Rating Scale (NRS, 0–10).

The clinical assessment was performed three times in total, through 1-week intervals and during the same time of day (late afternoon). The assignment of the individual participants to the old, middle-aged, or young red wine group has been changing from week to week to obtain *n* = 3 datasets per group.

**Informed consent:** Informed consent has been obtained from all individuals included in this study.**Ethical approval:** The research related to human use has been complied with all the relevant national regulations, institutional policies and in accordance with the tenets of the Helsinki Declaration and has been approved by the local institutional review board (protocol number 086/18, date of approval: 30th May 2018). The study was registered at the German Clinical Trials Register (DRKS) under the registration number DRKS00015062 before enrollment of research participants (date of registration: 9th July 2018).

#### 2.2.3 Blind tasting

Blind tasting, including wine experts and laypersons, was performed to determine the effect of storage time on the culinary aspects of red wine consumption. The expert group, which denoted itself “Les amateurs du Vin”, consisted of 3 former owners of a wine bar and shop (“Trocken und Trocken”, Cologne, Germany) with >30 years of experience in enology and wine tasting. In addition, seven wine laypersons were asked to participate in the tasting. All tasters were older than 18 years. The tasting was performed on a garden terrace during a mild summer evening to ensure relaxed though focused participants. CS, Ch, and Sh of each vintage were decanted into neutral carafes by an assistant who did not participate in the tasting. Carafes were given numbers from 1 to 9. Each varietal with its respective vintages was presented separately to the tasters in random order. Red wines were assessed using the University of California at Davis 20 Point Scale System (UC Davis-20). According to this scale, the following parameters are rated: clarity (2 points), color (2), bouquet (4), total acidity (1), sweetness (1), body/texture (2), flavor/taste (2), acescensy/bitterness (1), astringency (1), and overall quality (4). Items with less than 4 points are rated in steps of 0.5, and items with 4 points in steps of 1. Thus, a total score for each wine from 2 to 20 is possible. Only white bread and water were served to exclude any confounding factors on the tasting of the wines. All participants were instructed not to swallow but to spit out the test substances to keep a level head during the entire assessment period.

### 2.3 Statistical analysis

Statistical analysis and data visualization were performed using GraphPad PRISM 5 and MS Office 2010. Data are presented as mean values with either standard deviation (SD) or standard error of the mean (SEM), respectively. The significance of potential intergroup differences was tested using one-way ANOVA with the Newman–Keuls posthoc test. Different time points within a group were assessed using paired, two-tailed Student’s *t*-test, and *p* values <0.05 were considered statistically significant. The authors had full access to the entire dataset and fully analyzed; it is available from the corresponding author upon reasonable request.

## 3 Results

### 3.1 Aged red wines possess lower cytotoxic and pro-inflammatory potential with enhanced antioxidative properties *in vitro*

Nine different monovarietal wines (Cabernet Sauvignon [CS; vintages 2016, 2011 and 2006], Chianti [Ch; vintages 2014, 2009 and 2004], and Shiraz [Sh; vintages 2014, 2009 and 2004]) were analyzed for their biological effects. Aged varietals were significantly less acidic than younger ones (*p* < 0.005; Table 1) and contained more resveratrol (RVT), as evidenced by ELISA results for both Sh and CS wines (Table 1).

The Human hepatoma cells (HepG2 cell line) were incubated with CS, Ch, or Sh of the respective vintages for 24 h, and both cell supernatant and lysate were subsequently analyzed. While alanine aminotransferase (ALT) levels in the HepG2 cell supernatant were always below the detection limit, secretion levels of aspartate aminotransferase (AAT) and glutamate dehydrogenase (GLDH) decreased significantly after incubating hepatoma cells with old and middle-aged Ch and CS wines compared to the control (*p* < 0.005). In contrast, young vintages elicited a significant increase in both AAT and GLDH levels in the supernatant compared to the respective aged ones (*p* < 0.05). While neither old nor middle-aged wines affected IL-8 expression, as evidenced by RT-PCR, all young variants induced significant up-regulation by more than a factor of 4.5 (*p* < 0.005).

**Figure 1:**
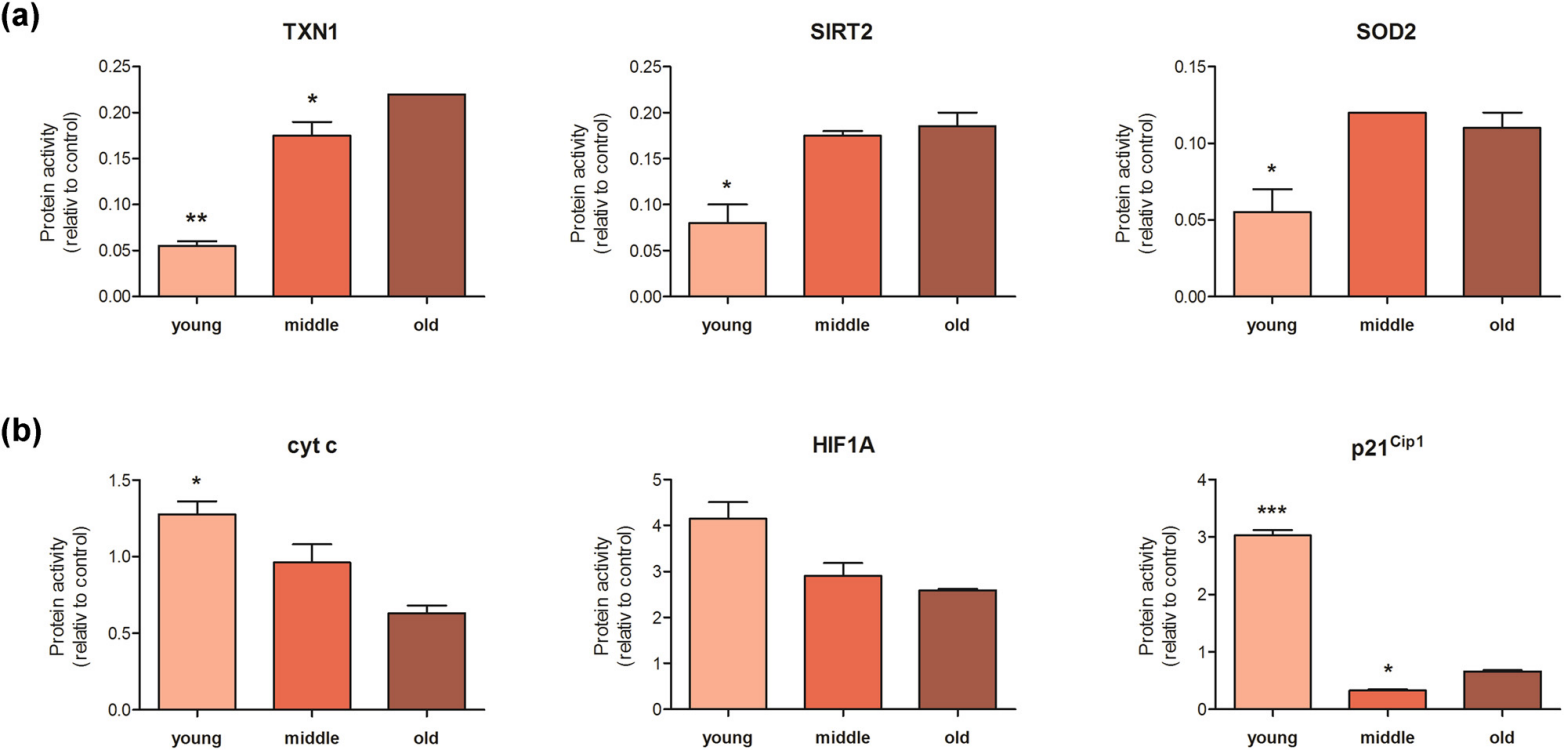
Impact of red wine age on cellular stress. Subconfluent HepG2 cells were incubated with young (light red), middle-aged (red), and old Shiraz (dark red), respectively (2.5 vol%, according to prior dose titrations). Culture medium was used as control. After 24 h, cells were lysed and total protein was extracted. Proteome profiling arrays were performed and analyzed according to the manufacturer’s instructions. The figure shows the protein activity, relative to control conditions. (a) Incubation of HepG2 cells with Shiraz reduces the protein activity of thioredoxin 1 (TXN1), sirtuin 2 (SIRT2), and superoxide dismutase 2 (SOD2) in an age-dependent manner. (b) Young wine increases protein activity of cytochrome c (cyt c), hypoxia-inducible factor 1-alpha (HIF1A), and p21^Cip1^, compared to incubation with middle-aged and old wine. Mean from replicates ± SEM; one-way ANOVA with Newman-Keuls posthoc test; *p < 0.05, **p < 0.01, ***p < 0.005 (vs old variant).

The impact of red wine age on cellular stress was assessed exemplary in Sh using array-based proteome profiling. Incubation of HepG2 cells in the presence of this varietal reduced the protein activity of thioredoxin 1 (TXN1), sirtuin 2 (SIRT2), and superoxide dismutase 2 (SOD2, Figure 1a). This was age-dependent, with younger wines leading to a significantly more pronounced reduction than the older ones. In contrast, these younger wines increased protein expression of cytochrome c (cyt c), hypoxia-inducible factor 1-alpha (HIF1A), and p21^Cip1^, as compared to the incubation with middle-aged and old wines (Figure 1b).

### 3.2 Young red wines increase both sympathetic activity and multitasking skills *in vivo* but aggravate post-degustation hangover severity

**Figure 2:**
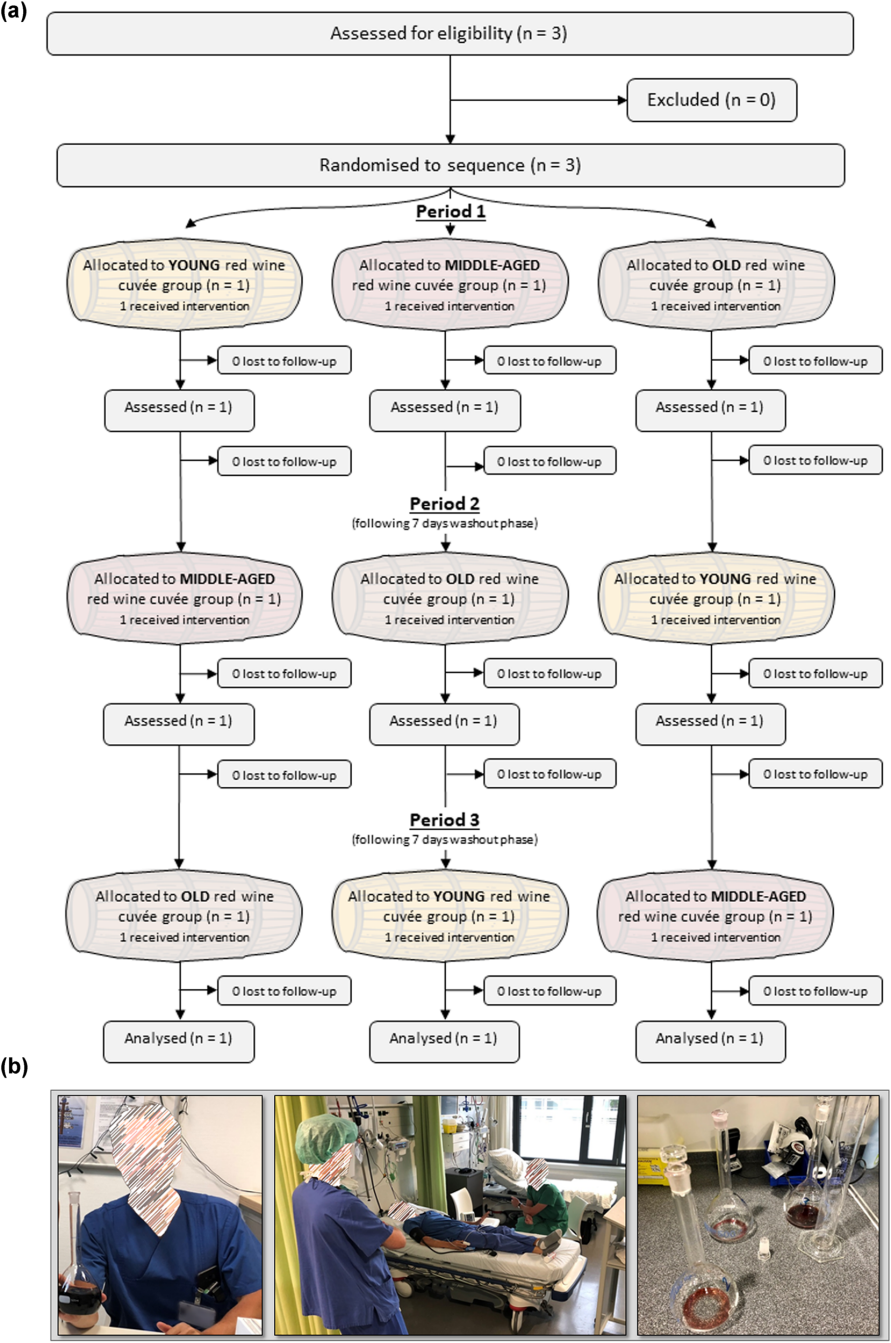
Clinical assessment of effects of red wine aging. (a) Effects of red wines of different ages were assessed in three healthy male volunteers using a randomized, blinded crossover design (see CONSORT flow chart [according to Dwan K, Li T, Altman DG, Elbourne D. CONSORT 2010 statement: extension to randomized crossover trials. BMJ. 2019 Jul 31;366:l4378.]). (b) CS, Ch, and Sh of the respective vintage were blended in equal proportions in round-bottom flasks (left). The image in the middle shows the experimental setup in the recovery room of the operation section (the person in the foreground is a neutral observer). Parameters were assessed with the participants lying in the supine position. Following baseline assessment, each participant had to drink 4.5 mL per kg bodyweight of old, middle-aged, or young red wine cuvée within 10 min, respectively (image on the right shows round-bottom flasks, emptied after ingestion of test fluids.

The short-term influence of red wine consumption on hemodynamics and sympathetic activity has been previously described [11]. Men are considerably more experienced with the effects and side-effects of alcohol consumption than women [6]. Therefore, the acute impact of drinking red wines of different vintages on clinical parameters was evaluated in male volunteers using a randomized, blinded crossover design. Hemodynamics, RVT metabolism, and mental performance were assessed both before and following ingestion of a standardized dosage of red wines of different vintages (Figure 2b).

Participants were blindly assigned to the young, middle-aged, or old wine group. Their heart rate slightly increased from 62 to 68 beats per minute 30 min following red wine ingestion in all groups (*p* < 0.05) with no differences observed among groups. In contrast, only test persons having consumed young wines experienced a significant short-term increase in mean arterial pressure from 87 ± 8.5 to 104 ± 8.1 mm Hg after 30 min after ingestion (*p* < 0.01), with subsequent normalization.

Average baseline (*T*_0_) RVT serum levels showed a high inter-individual variation, ranging from 0.65 ± 0.06 µg/mL (in participant 1, P1), through 1.32 ± 0.59 µg/mL (P2), up to 1.49 ± 0.41 µg/mL (P3). As expected from the analyses of different red wine vintages, RVT serum levels were altered most prominently by the ingestion of old wines (111 ± 37% 30 min after ingestion), compared to middle-aged (108 ± 27%) and young vintages (95 ± 13%). RVT metabolism varied as noticeably across participants as the baseline values did.

The participants had to perform three previously validated tests to assess both psychomotor coordination capabilities and vigilance level [12]. Young wine consumption significantly improved multitasking skills, based on the results of the ‘Divided Attention Test’ (DAT) 60 min post-consumption (mean trial duration at baseline 12.7 ± 8.0 s vs *T*_60_ 22.2 ± 11.9 s, *p* < 0.05). In contrast, old wines significantly reduced this maintenance among the contestants (mean performance at baseline 13.7 ± 6.5 s vs *T*_60_ 10.2 ± 5.4 s, *p* < 0.05). In the ‘Vigilance Test’ (VIG), participants’ performance was at a very high level already under the baseline conditions across all groups (92.4 ± 14.4% of skipped positions correctly detected). Red wine ingestion neither improved nor impaired participants’ performance in the VIG test (*T*_30_ 88.9 ± 14.5%, *T*_60_ 93.4 ± 8.2%). The same held for the ‘Digit Span Test’ (DST), a measure of working memory, which revealed no significant intergroup differences according to wine age.

Mean participants’ headache intensity on the day following assessment was rated 4/10 on the NRS (Numeric Rating Scale) for the young wines group (including the first-time-in-life migraine attack with NRS 7/10), compared to moderate NRS = 1/10 for both middle-aged and old wines.

### 3.3 Aged red wine variants perform better in terms of aroma and overall quality but worse in optical appearance

**Figure 3:**
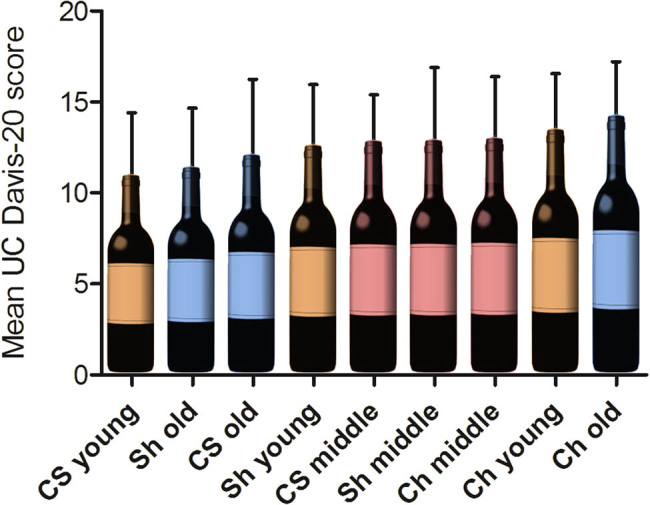
Effect of red wine age on culinary aspects. A blind tasting was performed including 3 wine experts and 7 laypersons. Cabernet Sauvignon (CS), Chianti (Ch), and Shiraz (Sh) of three different vintages were presented separately to the tasters in random order. Red wines were assessed using the University of California at Davis 20 Point Scale System (UC Davis-20), allowing a total score for each wine from 2 to 20. This figure shows test wines ranked according to their mean UC Davis-20 score. Orange (young), red (middle-aged), and blue (old) color indicates different vintages. Mean ± SD, *n* = 10.

The influence of storage on the culinary aspects of red wine was assessed by a standardized blind tasting using the University of California at Davis 20-Point Scale System (UC Davis-20). Having a cumulative mean UC Davis-20 score of 41 points, Ch was top-ranked, followed by Sh (37.2) and CS (36.3). Ch dominated the table with its three vintages, with the old vintage being the overall winner (Figure 3).

Middle-aged wines formed the midfield of the ranking, and both young and old vintages of CS and Sh brought up the rear. Young wines were significantly better accepted than either middle-aged or old vintages in terms of their optical appearance (clarity and color, *p* < .05). However, they were generally downgraded regarding aroma (bouquet and taste) and overall quality (Figure S1). Table 2 presents details on the scoring results of each wine.

**Table 2:** Averaged scoring results according to UC Davis-20 point scale

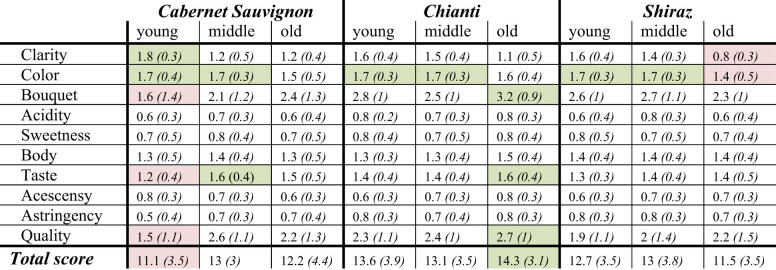

The respective best (green) and worst result (red) for optical appearance (composite parameter from clarity and color), bouquet, taste, overall quality, and total score are marked.

Mean ± SD, *n* = 10.

## 4 Discussion

Wine, unlike most other victuals, may improve by aging. The Gospel of Luke states that ‘… no one after drinking old wine wants the new, for they say, “The old is better.”’ (Luke 5:39). Nonetheless, anecdotal references to over-aging appear in newspapers, such as a report of a >150-year-old bottle of wine recovered from a shipwreck dating from the Civil War era. After opening, the fluid inside was found to taste like ‘… a mixture of crab water, gasoline, saltwater and vinegar …’ [14] – of course, this wine has indeed gotten (very) old, but without aging well. Surprisingly, to date, no study addressed the question of what aspects other than the culinary are likewise influenced by aging. For the first time, our results demonstrate a reduction in young wines’ pro-inflammatory and cytotoxic properties while enhancing both antioxidative and health-promoting features. Furthermore, aged wines seem to offer a superior experience regarding taste and overall quality and may be better tolerated.

Aging refers to the changes in wine composition after bottling (in contrast to maturation, which describes reactions in the wine following fermentation but before bottling). We found significant differences in the pH values of red wines of different vintages. Although the sensory qualities of red wine are influenced by far more than just acidity, studies on artificially modified pH values have shown that a higher pH (i.e., a lower acidity) usually promotes a finer and more desirable mouthfeel and sensory characteristic, compared to more acidic wines [15], which was reflected by the results of the UC Davis-20 scoring in our study. During winemaking, deacidification (i.e., the increase in pH) usually occurs due to the so-called secondary or malolactic fermentation (MLF), which is the decarboxylation of l-malic acid to l-lactic acid. Besides its impact on the wine’s acidity, other beneficial effects include the enhancement of sensory characteristics as well as microbiological stability [16], and MLF is therefore recommended for red wines today. Usually, MLF takes place during maturation and before bottling. Nevertheless, prolonged storage and aging in bottled red wines automatically result in increased pH values over time [17].

During aging, changes in wine aroma and color occur due to the polymerization of tannins and anthocyanins [5]. We found that aging, in general, can improve a wine in terms of sensory characteristics such as odor and taste, thus positively influencing the overall subjective impression of its quality. As the tasting experience varies individually, the differences between the vintages were not significant, though this should be expected with a larger sample size. It may also have clinical implications since the consumer may prefer an aged wine (that exhibits enhanced health-promoting characteristics) over a young one due to its improved culinary properties. In contrast, old wines suffer from a poor optical appearance. That need not necessarily indicate a reduced quality since aggregation and precipitation of wine’s compounds naturally occur during aging [5]. The type of bottle closure used may even worsen the perception of visual appearance.

Red wine is rich in RVT that possesses antioxidant, anti-inflammatory as well as anti-carcinogenic, and, particularly, anti-atherosclerotic properties [18,19]. Consequently, RVT in concentrated form is advertised as a lifestyle brand and an anti-aging product, sometimes even used as a face mask to make one’s pores feel ‘tighter’ [20]. Our results suggest that drinking aged red wines rather than young ones may be healthier due to higher RVT contents. However, although resveratrol may be quickly absorbed into systemic circulation within 30 min after intake [21], drinking more than 2,000 liters of red wine per day to achieve one’s desired RVT levels does not appear to be a suitable measure to fight aging [22,23]. We observed a wide inter-individual variation in the RVT metabolism that may reflect personal lifestyle and consumption habits. Participant 1 (P1), with a considerably lower baseline of the serum RVT level, compared to both P2 and P3, also showed a consistently more pronounced acute clinical reaction to the red wine ingestion in forms of personality changes by throwing paper balls at the other test persons or falling asleep spontaneously. Of note, this observation was independent of the wines’ age.

Of course, red wine contains several other components beyond RVT (phenolic acids, flavonols, flavonols, and anthocyanins) that also possess health-promoting properties and significantly influence its organoleptic characteristics. They are likewise considerably altered during the process of wine aging [5]. However, we decided to focus our analysis on RVT as it is, on the one hand, the most studied and best-described stilbene [24] and, on the other hand, it is considered the most effective red wine component in terms of health-promoting effects due to its distinct antioxidant properties [25].

As the liver is known to be affected by red wine consumption, we used a stable hepatoma cell line to assess the biological effects of different vintages of red wines. Aminotransferases and GLDH are well-known clinical parameters of liver damage. However, the direct influence of red wine on their serum levels remains unclear. Even in people older than 100 years and consuming up to 500 mL red wine per day, no increase in aminotransferases has been observed [26]. This may also be applied to other alcoholic beverages [27]. Our experiments revealed an increase in both AAT and GLDH levels after incubating hepatoma cells with young red wine vintages, which may indicate its increased cytotoxicity [28]. Proteome profiling, as well as RT-PCR results, support this assumption. TXN1, SOD2, and SIRT2 have an outstanding role in reducing hepatic oxidative stress and mitigating mitochondrial dysfunction [29]. In our study, wines of young age most prominently reduced these cytoprotective enzymes. On the other hand, they elicited a clear increase in the activity of pro-inflammatory, pro-apoptotic, and pro-fibrotic cell stress-signal proteins IL-8, cyt *c*, HIF1A, and p21^Cip1^ [30–32]. The profile of red wines’ microconstituents and in particular polyphenols such as RVT has been shown to exert anti-inflammatory properties, and altered RVT content in red wine due to storage and aging may explain differential modulation of cytoprotective and -toxic proteins in human hepatoma cells in our experiments [5,33,34].

Alcohol promotes various acute clinical effects. Even gastric emptying was found affected by wine or cherry schnapps consumed together with Swiss cheese fondue [35]. However, the most prominent is its influence on neurocognitive skills [36]. Surprisingly, in the DAT (measuring multitasking skills), the participants performed almost twice better after consuming young red wine, while, in contrast, these skills weakened after having drunk old wine. Wong et al. demonstrated that acute RVT intake improved multitasking skills in Type 2 Diabetes mellitus patients within 1–2 h and that neurocognitive performance correlated with RVT plasma levels [37]. However, given increased RVT content in aged compared to young wines in our study, this phenomenon may not explain improved performance in the DAT following consumption of young red wine in our experiments. Tyrosine, an amino acid found in red wine and whose content is likewise altered by aging due to decarboxylation to tyramine [38], has also been shown to improve multitasking skills within a short time after consumption which may furthermore explain our results [39].

Activities demanding high levels of multitasking, such as driving a car, must be avoided after alcohol (and particular old wine) consumption. However, in rare cases, drinking might even improve driving skills, at least if you are a famous MI6 agent engaged in a high-speed car chase after consuming more than 10 double vodka martinis (shaken, not stirred!) [40]. How interesting it would be to perform the DAT with female participants who are supposed to be better at multitasking than sober men! [41].

In our study, red wine did not influence vigilance. This was, in fact, not at all that surprising since all candidates were board-certified anesthesiologists who are, although always said to be on a break [42], accustomed to being highly vigilant all the time and to fighting boredom during day-consuming cases in the operation room [43]. Thus, they performed almost perfectly already at the baseline with no possibility for further improvement.

Sympathetic stimulation, together with a severe headache and even migraine the next day, might create the impression of ‘young and wild’ wines, making you regret the following morning for boozing all night. Besides the fact that hangover severity depends on the one hand on the kind of liquor being drunk [44], on the other hand, its age may have a further impact when it comes to red wine. The latter is rich in biogenic amines (BA) which are formed from amino acids by decarboxylation [38]. Esposito et al. demonstrated that BA levels were much higher in wines on tap than in those aged in bottles and that consumption of BA-rich wines triggered severe clinical symptoms, including headache, flushing, nausea, and increased blood pressure and heart rate, underlining our results [45]. In addition, the consumer’s age may likewise influence hangover severity, suggesting younger people are better advised to drink aged wines [46].

Our study yet has limitations. Although purchased from professional wine retailers, proper storage conditions of test substances from the date of production can only be assumed. Moreover, varying viticultural factors as well as specific farming techniques employed during the production years may influence the chemical composition of the raw material and consequently the study results. Red wines from only one vintage would have had to be stored for years with intermediate assessments to eliminate these potential confounders. However, the ageing of test persons might influence the results as well. Furthermore, thirsty members of the laboratory crew could eliminate the test substances over the years.

Our report ignores a number of other bioactive red wine components such as anthocyanins and polyphenols beyond resveratrol which also have a significant impact on its organoleptic and health-promoting properties. This limitation has already been addressed and explained earlier.

Our results are not necessarily transferrable to cheaper red wines. All the test substances were from the mid-price segment, as a cheap red wine is usually produced to be consumed immediately and not to be stored for years. Whether our data may be transferred to alcohol-free red wines (taking into account very recent research results [6]) remains to be evaluated.

Although participants of the blind tasting were instructed to spit out the liquid, this was not strictly controlled. As a result, increased blood alcohol content (BAC) may have influenced their rating. Normalization of results to the measured BAC could be one way to deal with this issue, but this was not feasible in this study.

Of course, the limited number of participating volunteers in the clinical part of the study is even more critical, and different diets may also have influenced the results of our observation. Therefore, the results do not necessarily have to be representative and should be re-evaluated by others in a larger cohort.

**Figure 4:**
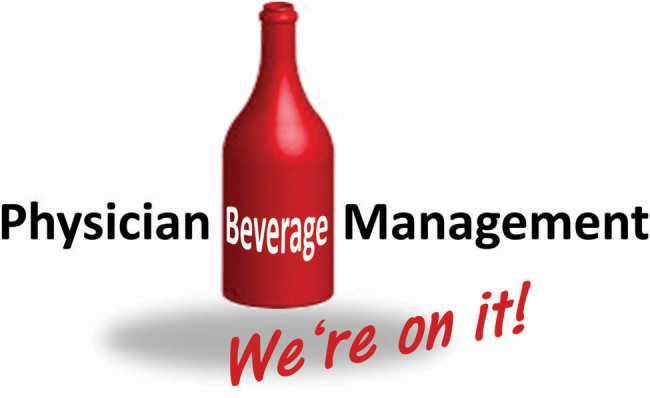
Proposed logo for the *Physician Beverage Management* (PBM) program. For the iconic logo of the worldwide *Patient Blood Management* (PBM) program, please refer to www.patientbloodmanagement.de.

Taken together, we have no evidence for the existence of a *red wine storage lesion*. On the contrary, our study demonstrates that red wine can improve in quality by storage and aging in several ways. Of particular importance is to consider the relevant requirements that of course have to follow strict rules and are regulated by law as long as they relate to production and commercial long-term storage of red wine (e.g., the European Union Common Agricultural Policy [CAP] Wine Regime [47] or the United States Alcohol and Tobacco Tax and Trade Bureau [TTB] [48]). However, since connoisseurs and critical and perioperative care wine enthusiast increasingly tend to store their fine liquids in own home-based cellars, we plead for consensus-based guidelines for this purpose, as it is common in clinical medicine. Therefore, and to follow the concept of patient safety, we recommend to initiate the *Physician Beverage Management* program, of which the acronym (PBM) and logo (Figure 4) are a reminiscence of and a tribute to *Patient Blood Management*, the globally known initiative for perioperative quality improvement [2].
